# Gas-solid fluidization modification of calcium carbonate for high-performance poly (butylene adipate-co-terephthalate) (PBAT) composites

**DOI:** 10.3389/fchem.2022.1119978

**Published:** 2023-01-12

**Authors:** Jinzhi Shang, Chang Li, Yang Song, Mingkai Yan, Lin Li, Chaoquan Hu

**Affiliations:** ^1^ School of Chemical Engineering, Shenyang University of Chemical Technology, Shenyang, Liaoning, China; ^2^ Nanjing IPE Institute of Green Manufacturing Industry, Nanjing, Jiangsu, China; ^3^ State Key Laboratory of Multiphase Complex Systems, Institute of Process Engineering, Chinese Academy of Sciences, Beijing, China

**Keywords:** gas—solid fluidization, chemical modification, biodegradable composites, rheological properties, mechanical properties

## Abstract

Modifying biodegradable poly (butylene adipate-co-terephthalate) (PBAT) plastic with inorganic fillers is critical for improving its overall performance, lowering the costs, and expanding its application scope. The chemical modification method for the inorganic filler determines the application performance of PBAT composites. In this work, gas—solid fluidization method was developed as a simple, efficient, and scalable strategy for chemically modifying CaCO_3_ filler. The modified CaCO_3_ filler was mixed with PBAT and melt extruded to prepare biodegradable PBAT/CaCO_3_ composites. The characterization results show that gas—solid fluidization method combines the traditional wet modification method’s excellent modification effect with the scalability of the traditional dry modification method. The effects of modification methods and amount of CaCO_3_ filling on the crystallinity, mechanical, and rheological properties of PBAT/CaCO_3_ composites were compared. The results demonstrated that PBAT/CaCO_3_ composites containing 30% gas—solid fluidization modified CaCO_3_ could still maintain excellent overall performance. As a result, this work provides a simple, efficient, and scalable method for chemically modifying inorganic fillers and preparing biodegradable composites.

## 1 Introduction

The widespread application of traditional petroleum-based plastics has resulted in serious environmental pollution issues. As a result, countries worldwide have enacted laws and regulations to encourage the use of biodegradable materials (Madhavan Nampoothiri et al., 2010; Kasirajan and Ngouajio, 2012); however, the cost of most biodegradable plastics is 2–4 times higher than that of traditional non-biodegradable materials, thereby making market adoption difficult. Therefore, developing low-cost, high-performance biodegradable materials is a tried-and-true method for reducing the environmental problems caused by waste plastics. Poly (butylene adipate-co-terephthalate) (PBAT) is one of the most promising environment-friendly materials to replace conventional plastics because it combines the ductility of aliphatic polyesters with the mechanical strength of aromatic polyesters (Biundo et al., 2016; Camani et al., 2021; Zhang et al., 2021); however, its low modulus, as well as poor gas barrier properties, low crystallinity, and high cost, have hampered its further development and application (Cavalcanti et al., 2007; Zhai et al., 2020; Wei et al., 2021). Therefore, modifying PBAT with functional materials is critical for improving its overall performance and lowering application costs.

Inorganic fillers with abundant sources and low prices are frequently used for the co-blending modification of PBAT (Mondal et al., 2014; Mukhopadhyay et al., 2015; Li et al., 2018; Xing et al., 2019), with CaCO_3_ being one of the most commonly used fillers in the polymer industry (Kemal et al., 2013; Mantia et al., 2013; Tang et al., 2014; Zapata et al., 2018); however, CaCO_3_ is a hydrophilic inorganic filler with poor interfacial bonding ability with the hydrophobic PBAT matrix, necessitating chemical functionalization treatment to modify its surface properties (Chen et al., 2004). Fatty acid salt modification (Wang et al., 2007; Jeong et al., 2009; Tran et al., 2010), phosphate ester modification (Sheng et al., 2004), coupling agent modification (Doufnoune et al., 2003; Li et al., 2019), surface polymer grafting modification (Han and Kim, 2012), and *in situ* polymerization (Li et al., 2021) are the most common chemical modification methods for inorganic fillers. Among these, coupling agent modification has the advantages of simplicity and low cost.

Titanate is a common coupling agent that, when surface functionalized, can significantly improve CaCO_3_ hydrophobicity (Wang et al., 2010). When compared with silane coupling agents, titanate coupling agent modified CaCO_3_ has better interfacial interactions with the polymer matrix, resulting in a more significant improvement in composite performance (Upadhyaya et al., 2012). Depending on the treatment method, surface functionalization methods for inorganic fillers are classified as dry or wet (Shi et al., 2006). Tran et al. (2010), for example, synthesized stearic acid-modified CaCO_3_ in Ca(OH)_2_ solution *in situ* and discovered that the corresponding water contact angle could reach 127.5°. In contrast, the water contact angle for dry-method-modified CaCO_3_ was only 110.3° (Jeong et al., 2009). As a result, wet modification method with a homogeneous treatment has a better functionalization effect than dry modification; however, it consumes a large amount of solvent and produces a significant amount of liquid waste, making it unsuitable for industrial-scale production. Therefore, it is highly desirable to develop a new chemical modification method that combines the benefits of dry and wet modification methods.

The gas—solid fluidization method was developed as a simple, efficient, and scalable strategy for chemically modifying CaCO_3_ filler. Additionally, the physicochemical properties of CaCO_3_ modified by gas—solid fluidization were compared with those of dry- and wet-modified products; the results confirmed this method’s excellent modification effect. The effects of surface modification methods and CaCO_3_ content on the crystalline properties, mechanical strength, and rheological performance of PBAT/CaCO_3_ composites were also investigated. The modification to gas—solid fluidization could significantly improve the interfacial compatibility between inorganic filler and polymer matrix, thereby laying the groundwork for large-scale preparation and application of high-performance biodegradable composites.

## 2 Experimental

### 2.1 Materials

PBAT (TH801T) pellets were provided by Xinjiang Lanshan Tunhe Polyester Co. (China). The PBAT samples had a melt flow index of 3.0–5.0 g/10 min (2.16 kg at 190°C), density of 1.20–1.28 g/cm^3^, and melting point of 110–120°C. The commercial CaCO_3_ powder was purchased from Lingshou Zhanteng Mineral Products Processing Plant (Hebei, China). Titanate was supplied by Shanghai Macklin Biochemical Technology Co. (China). And liquid paraffin was provided by Sinopharm Chemical Reagent Co. (Shanghai, China).

### 2.2 Surface modification of CaCO_3_

**FIGURE 1:**
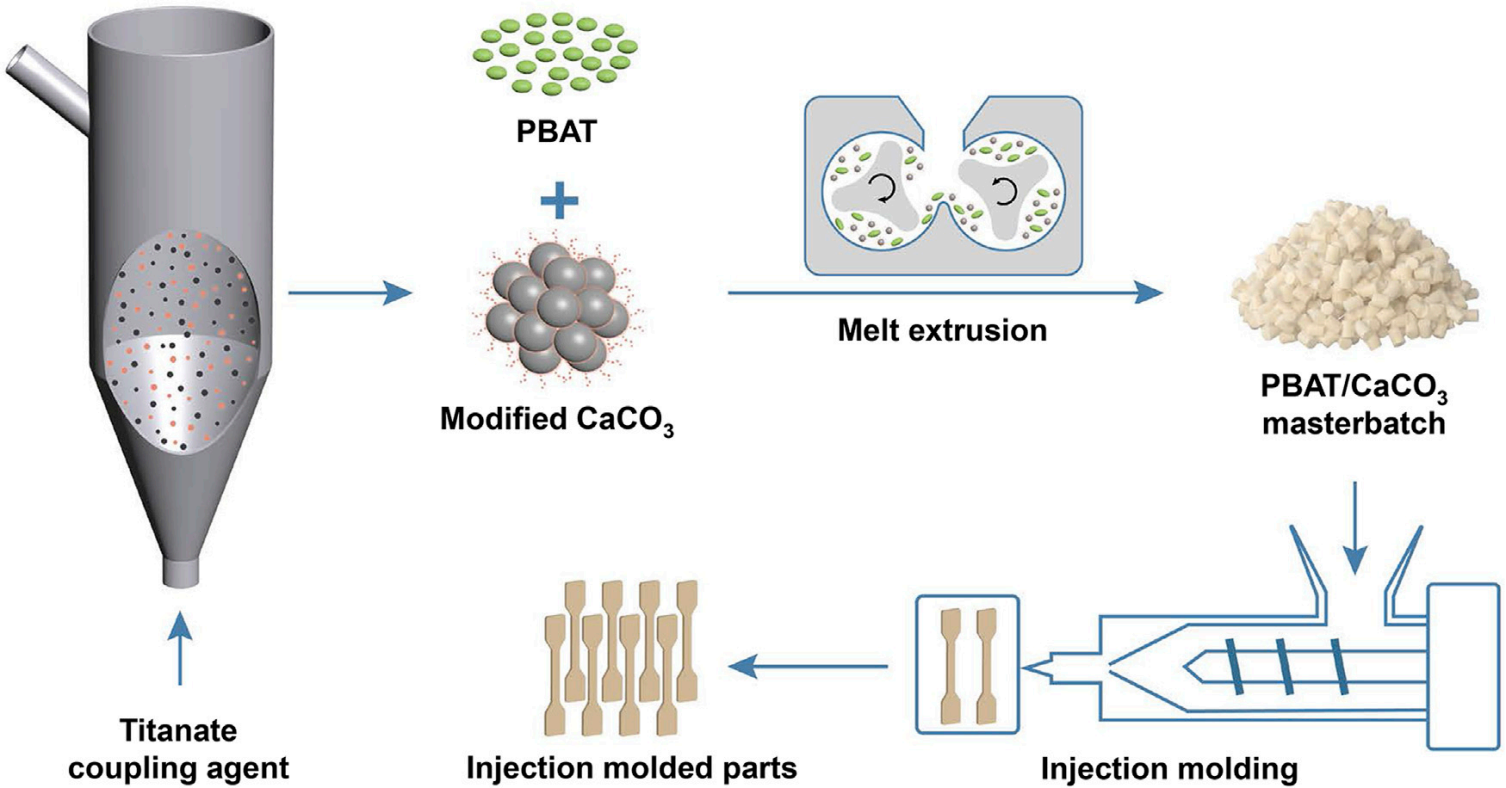
Schematic diagram for the preparation of gas—solid fluidization modified CaCO_3_ and the corresponding PBAT/CaCO_3_ composite.

The pristine CaCO_3_ powder was dried at 80°C for 12 h before surface modification treatment to remove surface moisture. CaCO_3_ was gas—solid fluidized and modified by adding it into a glass fluidization reactor; 2.5% titanate coupling agent was added in the atomizing cup and mixed with liquid paraffin in a 1:1 mass ratio. The fluidization reactor was then placed in a vertical tube furnace. Next, the titanate coupling agent and liquid paraffin were atomized by an air compressor and reacted with CaCO_3_ at 60°C for 30 min (Figure 1); for the dry modification of CaCO_3_, they were then added to CaCO_3_ powder and reacted in a high-speed stirrer at 60°C for 30 min. Whereas, for the wet modification method, CaCO_3_ was dispersed into anhydrous ethanol (mass ratio 1:5), and then 2.5 wt.% titanate coupling agent and liquid paraffin were added into it and stirred at 60°C for 30 min. This sample was then filtered and dried at 80°C for 12 h.

### 2.3 Preparation of PBAT/CaCO_3_ composites

PBAT and CaCO_3_ in various mass ratios (Table 1) were mixed in a high-speed mixer before being added into a twin-screw extruder for blending and melt-extrusion, air-cooling, and pelletizing (Figure 1); the melting temperature was 160°C, and the extrusion rate was 90 rpm. The obtained pellet samples were dried at 80°C for 12 h to produce PBAT/CaCO_3_ composites. In addition, sample strips were created by extruding homogeneous pelletized blends through an injection molding machine in accordance with national standards (GB/T22554-2006) for tensile performance tests.

**TABLE 1:** The composition of PBAT/CaCO_3_ materials prepared in this study.

Samples	PBAT (wt.%)	CaCO_3_(wt.%)			
		Unmodified (u)	Dry (d)	Wet (w)	Gas-solid fluidization (f)
PBAT	100	—	—	—	—
P9C1-u	90	10	—	—	—
P8C2-u	80	20	—	—	—
P7C3-u	70	30	—	—	—
P6C4-u	60	40	—	—	—
P9C1-d	90	—	10	—	—
P8C2-d	80	—	20	—	—
P7C3-d	70	—	30	—	—
P6C4-d	60	—	40	—	—
P9C1-w	90	—	—	10	—
P8C2-w	80	—	—	20	—
P7C3-w	70	—	—	30	—
P6C4-w	60	—	—	40	—
P9C1-f	90	—	—	—	10
P8C2-f	80	—	—	—	20
P7C3-f	70	—	—	—	30
P6C4-f	60	—	—	—	40

## 3 Results and discussion

### 3.1 Characterization of surface-modified CaCO_3_

#### 3.1.1 Infrared spectral analysis

**FIGURE 2:**
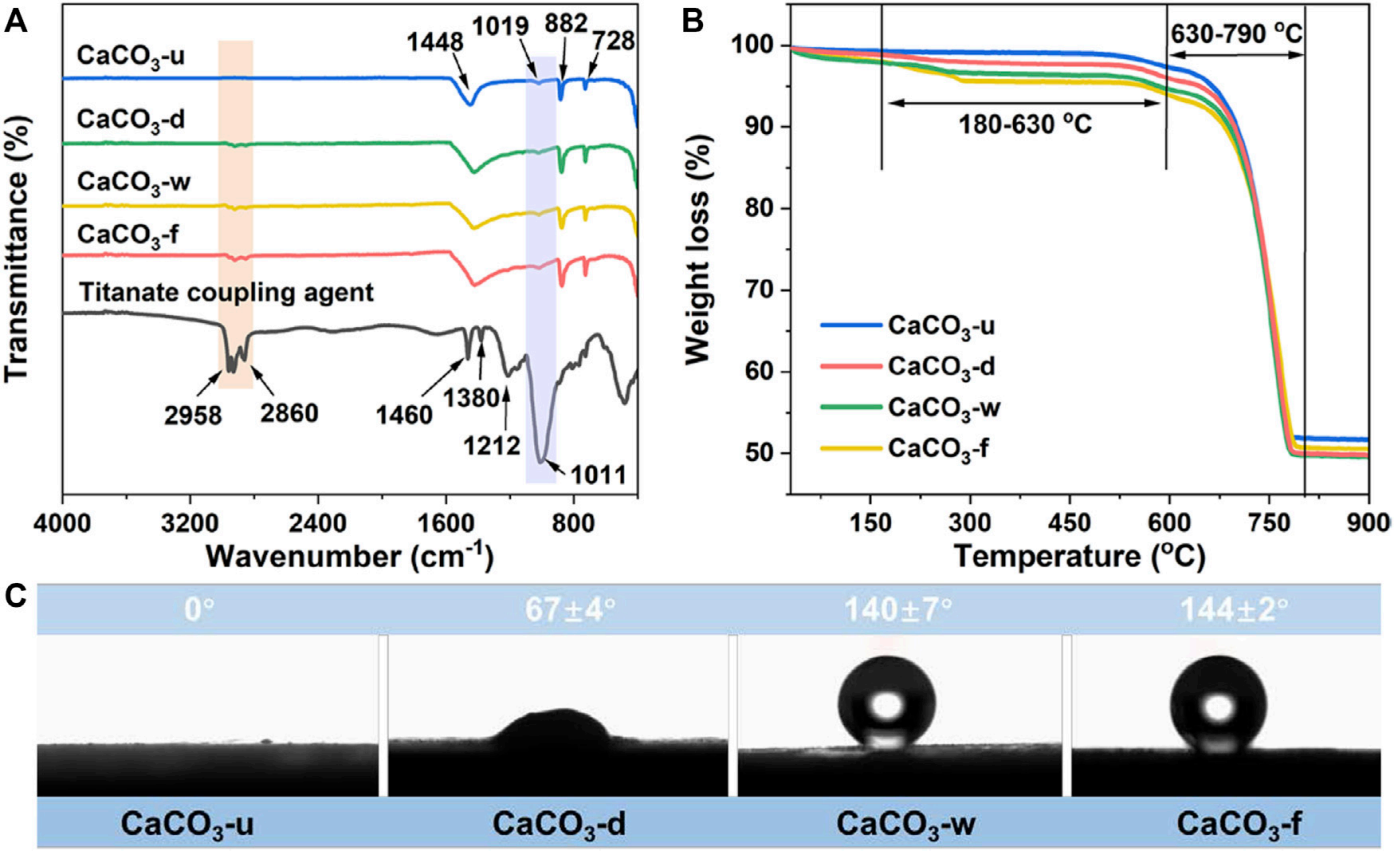
**(A)** FT-IR spectroscopy, **(B)** TGA, and **(C)** Water contact angle of CaCO_3_ samples prepared by different modification methods.

The surface chemical composition of the modified CaCO_3_ particles was studied using Fourier transform infrared (FT-IR) spectroscopy (Wang et al., 2010; Tang et al., 2014; Li et al., 2019); the results are shown in Figure 2A. The characteristic signals of unmodified CaCO_3_ particles are O–C–O in-plane bending vibration (728 cm^−1^), O–C–O out-of-plane bending vibration (882 cm^−1^), C–O asymmetric stretching vibration (1,019 cm^−1^), and C–O symmetric stretching vibration (1,448 cm^−1^). As compared to unmodified CaCO_3_, modified CaCO_3_ (CaCO_3_-d, CaCO_3_-w, and CaCO_3_-f) has characteristic absorption peaks of–CH_3_ (2,958 cm^−1^) and–CH_2_– (2,860 cm^−1^) stretching vibration. Meanwhile, compared with dry- and wet-modified CaCO_3_, the gas-solid fluidization modified product has stronger absorption peaks for–CH_3_ and–CH_2_– stretching vibration, verifying its excellent functionalization effect. These findings indicate that the long-chain alkyl groups of the titanate coupling agent have been encapsulated on the surface of CaCO_3_ particles. In addition, the modified CaCO_3_ particles exhibit a distinct absorption peak for C–Ti–O–CaCO_3_ at 1,024 cm^−1^, indicating that the surface groups of CaCO_3_ is converted from–OH to C–Ti–O–CaCO_3_ and the coupling agent is tightly adsorbed on the CaCO_3_ particle surface.

#### 3.1.2 Thermogravimetric analysis

Thermal stability is an important parameter that could quantitatively reflect the amount of modification of coupling agent molecules (Tang et al., 2014); therefore, thermogravimetric analysis (TGA) was performed on CaCO_3_ particles before and after modification. The modified CaCO_3_ showed a progressive mass loss in the temperature range of 180°C–630°C due to the decomposition of titanate coupling and liquid paraffin attached to the CaCO_3_ particle surface (Figure 2B). Following that, a second mass loss occurs in the temperature range of 630°C–790°C due to the decomposition of CaCO_3_ into CaO and CO_2_. The mass loss of gas–solid fluidization modified CaCO_3_ is 5.27% in the temperature range of 180°C–630°C, which is higher than that of unmodified CaCO_3_ (.42%), dry-modified CaCO_3_ (2.85%), and wet-modified CaCO_3_ (4.83%). According to these findings, the gas–solid fluidization modification allows more titanate coupling agents to coat the surface of CaCO_3_.

#### 3.1.3 Contact angle analysis

CaCO_3_ surface properties are crucial in determining its interfacial compatibility with polymer matrix (Jeong et al., 2009; Tran et al., 2010; Titone et al., 2020); therefore, the contact angles of unmodified and modified CaCO_3_ samples were tested to characterize hydrophobicity variation, and the results are shown in Figure 2C. Water droplets penetrate the powder sample so quickly that the observed contact angle is 0° because the unmodified CaCO_3_ is highly hydrophilic. The modification of titanate coupling agent treatment significantly increased the CaCO_3_ hydrophobicity, with corresponding contact angles increasing to 67°, 140°, and 144° for dry-, wet-, and gas–solid fluidization modified samples, respectively. CaCO_3_’s conversion from hydrophilic to lipophilic reduces its surface energy and improves the interfacial compatibility between the inorganic filler and polymer matrix. Notably, the hydrophobicity of CaCO_3_ modified by gas—solid fluidization is higher than that of dry and wet modification methods, indicating that the atomized coupling agent molecules could fully react with CaCO_3_ particles to form abundant chemical bonds.

### 3.2 Characterization of PBAT/CaCO_3_ composites

#### 3.2.1 Crystallinity analysis

**FIGURE 3:**
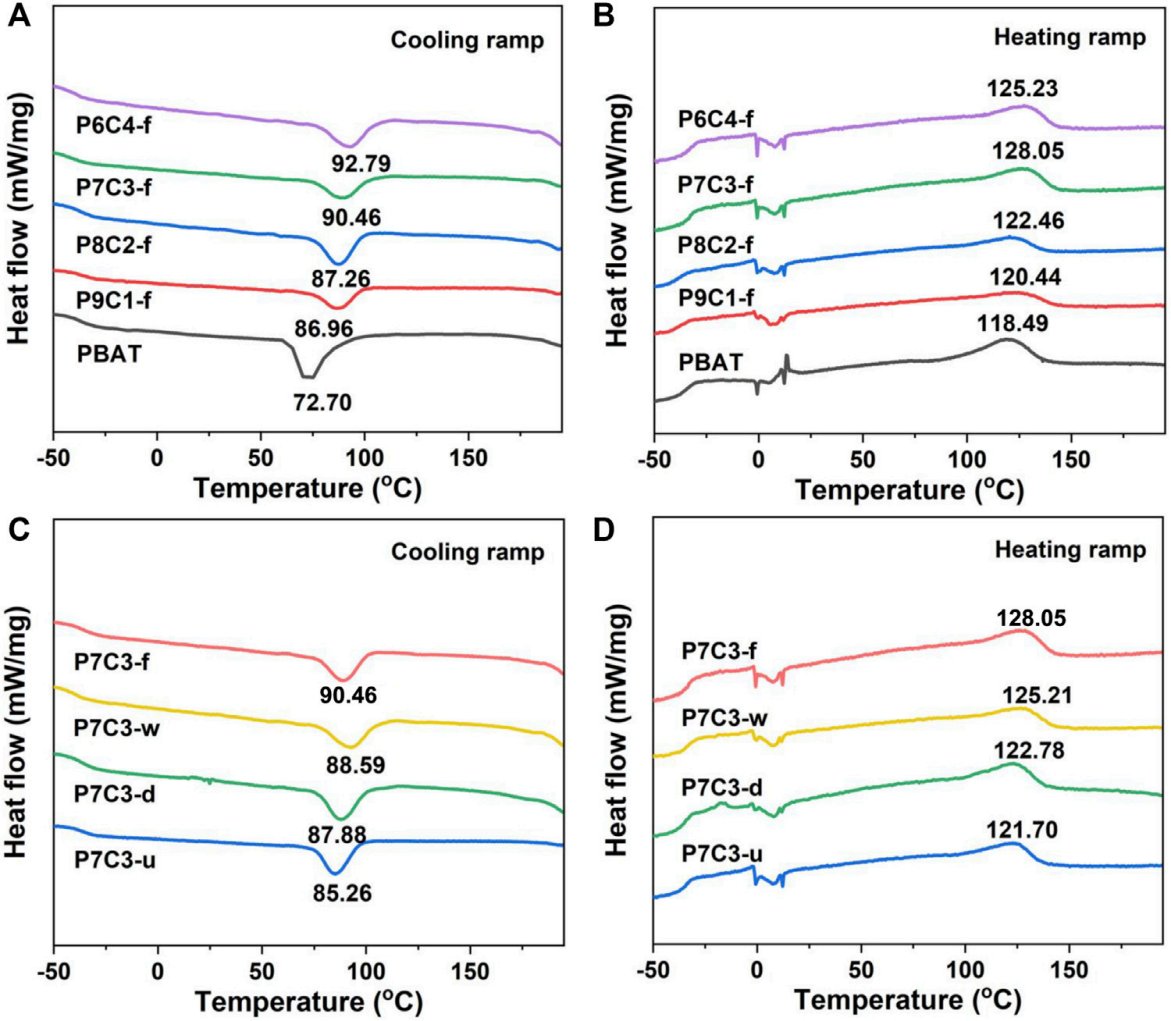
**(A)** Cooling and **(B)** heating ramps of PBAT composites filled with different amounts of gas–solid fluidization modified CaCO_3_. **(C)** Cooling and **(D)** heating ramps of PBAT composites filled with 30 wt.% CaCO_3_ modified by different methods.

Because the crystallization and melting temperatures of the composite could directly reflect the interfacial compatibility between the inorganic filler and polymer matrix, differential scanning calorimetry analysis was performed to investigate the relevant parameters. The crystallization and melting temperatures of PBAT composites filled with different amounts of fluidization modified CaCO_3_ are shown in Figures 3A, B. The crystallization temperature of PBAT composites increases with the addition of CaCO_3_ (Figure 3A); this can be attributed to the formation of a coupling between CaCO_3_ and the PBAT matrix, which acts as a nucleus site for heterogeneous nucleation and causes PBAT molecules to crystallize further. For the complete melting of CaCO_3_-filled PBAT, higher enthalpy and temperature are required. As shown in Figure 3B, the melting temperature of PBAT composites increases and then decreases as the amount of added CaCO_3_ increases. When the CaCO_3_ content is 30 wt.%, the destructive effect on the composite material is very small, resulting in crystallization and a higher melting temperature; however, when the added content reaches 40 wt.%, the crystallinity rapidly decreases because the excessive content has a destructive effect on crystallization, resulting in a gradual decrease in the degree of crystallization of the composites. Furthermore, the higher the filler content is, the more obvious is the agglomeration phenomenon, which impedes molecular chain movement and makes the material less crystalline.

Figures 3C, D show that the melting and crystallization temperatures of composites filled with 30 wt.% CaCO_3_ using various modified methods differ. The crystallinity of P7C3-u, P7C3-d, P7C3-w, and P7C3-f was calculated to be 26.98%, 28.22%, 33.01%, and 33.39%, respectively (Table 2). The crystallinity of P7C3-d is lower than that of P7C3-w and P7C3-f due to the poor nucleation ability of PBAT composites. It is demonstrated that as the crystallinity of the polymer matrix increases, the composites will have higher modulus, better thermal stability, and higher strength. As a result, the PBAT composites containing gas—solid fluidization modified CaCO_3_ have high crystallinity and effectively improve the interfacial compatibility between the filler and matrix molecules.

**TABLE 2:** DSC crystallization parameters of PBAT/CaCO_3_ composites.

Samples	Tc (°C)	Tm (°C)	ΔHm (J/g)	Χc (%)
P7C3-u	85.26	121.70	43.94	26.98
P7C3-d	87.88	122.78	45.96	28.22
P7C3-w	88.59	125.21	53.75	33.01
P7C3-f	90.46	128.05	54.38	33.39

The degree of crystallinity (Χ_c_) is estimated from ΔH_m_/ΔH_m_^0^ ω_PBAT_ × 100%, where ΔH_m_ is the fusion heat of the sample, ΔH_m_^0^ is the melting enthalpy that equal 114J/g for PBAT, and ω_PBAT_ is the mass content of PBAT in the composite.

#### 3.2.2 Rheological properties

**FIGURE 4:**
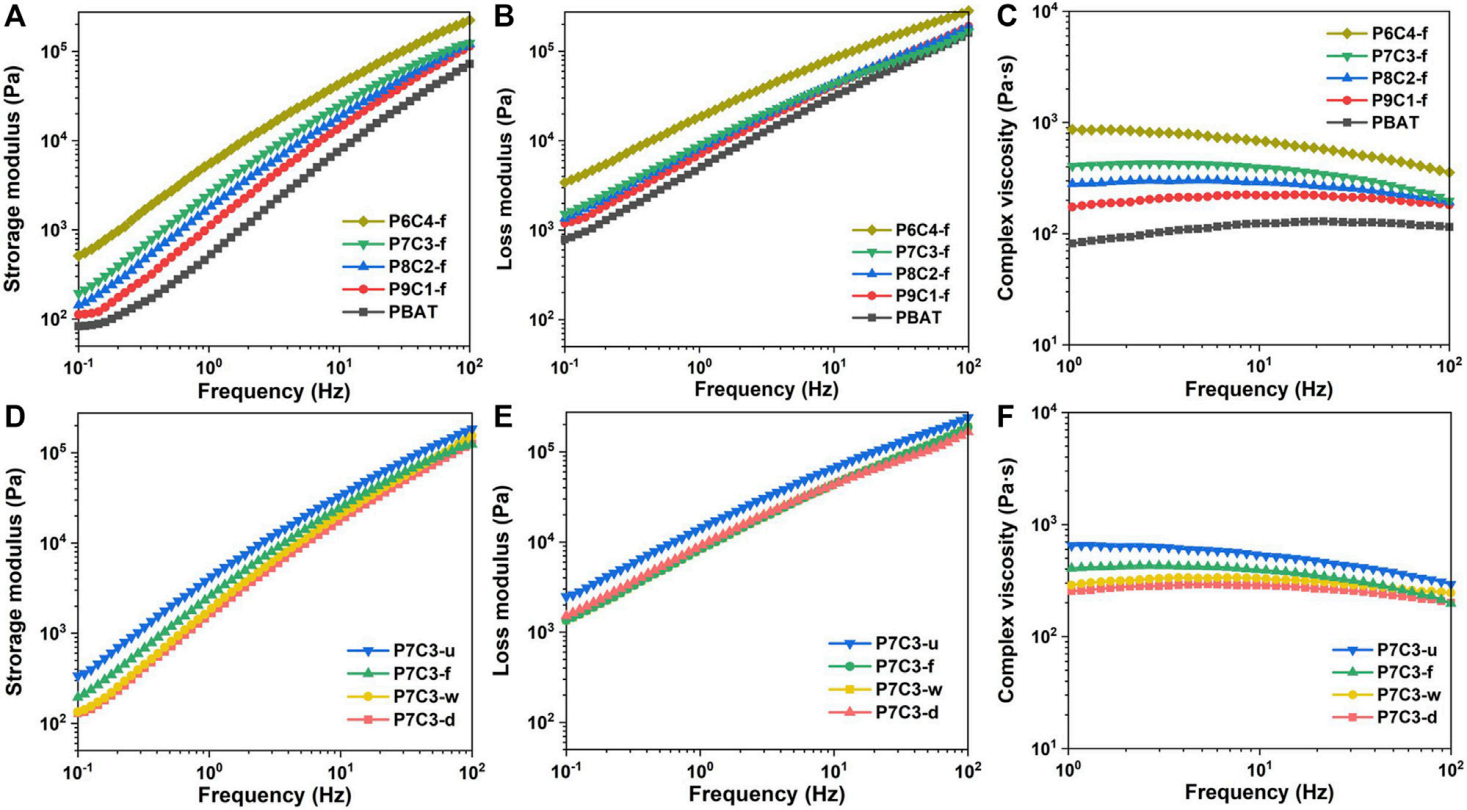
**(A)** Storage modulus, **(B)** loss modulus, and **(C)** complex viscosities of PBAT composites filled with different content of gas–solid fluidization modified CaCO_3_. **(D)** Storage modulus, **(E)** loss modulus, and **(F)** complex viscosities of PBAT composites filled with 30 wt.% CaCO_3_ obtained by different modification methods.

The rheological properties of PBAT/CaCO_3_ composites were investigated to determine the dispersibility of inorganic filler in the polymer matrix and intermolecular interactions. Figures 4A–C depict the frequency dependence of PBAT composites filled with various amounts of gas—solid fluidized modified CaCO_3_. Figure 4A shows that the storage modulus (G′) of PBAT composites increases with increasing frequency, which can be attributed to the chain segment of PBAT macromolecules being unable to keep up with the shear rate and thus resisting the shear stress, resulting in a greater resistance with higher frequency. Furthermore, the G′ values increase as the CaCO_3_ content increases because the addition of CaCO_3_ improves the interaction of the blended system and requires more energy for deformation. Figure 4B depicts the variation of the loss modulus (G″) with the addition of gas–solid fluidization modified CaCO_3_. The G″ values, for both pure PBAT and PBAT/CaCO_3_ composites, increase as frequency increases. As frequency increases, so does intermolecular friction and loss capacity, resulting in a higher loss modulus. The interactions between PBAT macromolecular chains and CaCO_3_ particles play a decisive role in the intermolecular friction in PBAT composites filled with varying amounts of CaCO_3_. As a result, the increased CaCO_3_ causes higher energy loss due to friction. Figure 4C depicts the change in complex viscosity with the addition of gas—solid fluidization modified CaCO_3_. The PBAT/CaCO_3_ composite viscosity is higher in the low-frequency region than that in the high-frequency region, indicating shear thinning behavior. Meanwhile, the PBAT/CaCO_3_ composite viscosity increases with filler content, indicating that the chemical modification treatment significantly increases intermolecular fusion between PBAT and CaCO_3_, thereby impeding molecular chain movement.

Figures 4D, E show the variation of storage and loss modulus with frequency for composites filled with 30 wt.% CaCO_3_ and modified in various ways. Chemical modification treatment reduces the mobility of molecule chains, resulting in a longer relaxation time and lower energy storage modulus when compared to an unmodified PBAT/CaCO_3_ composite. Figure 4F depicts the variation in viscosity with frequency for composites filled with various concentrations of modified CaCO_3_. The modified CaCO_3_-filled composites have a higher viscosity than the unmodified ones due to improved interfacial compatibility, which facilitates intermolecular movement and, thus, reduces viscosity. Furthermore, the PBAT composite filled with gas—solid fluidization modified CaCO_3_ has the lowest viscosity, indicating that the coupling agent and PBAT molecular chains are effectively cross-linked.

#### 3.2.3 Mechanical strength

**FIGURE 5:**
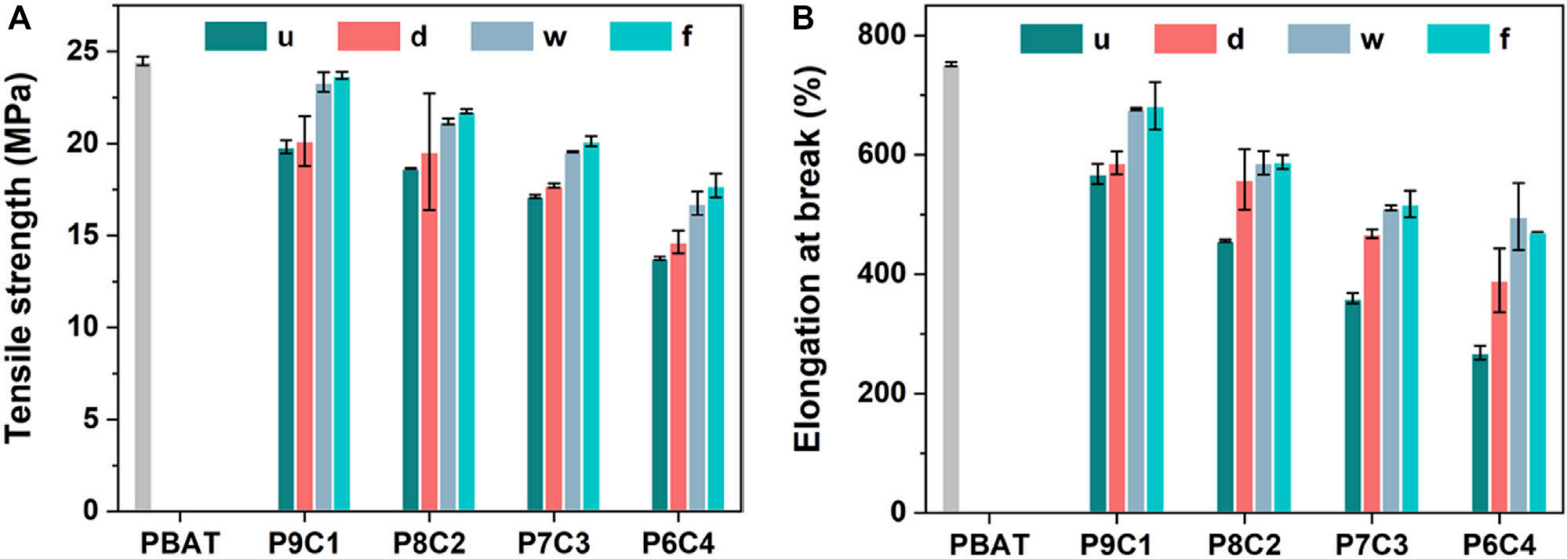
**(A)** Tensile strength and **(B)** elongation at break of PBAT composites filled with different content and modification method of CaCO_3_.

Tensile strength and elongation at break were used to evaluate the mechanical properties of the composites for determining the effect of filler modification methods and content on them. Figure 5 depicts the results. Overall, the order of tensile strength and elongation at break is PBAT > P9C1 > P8C2 > P7C3 > P6C4 because in the blended system, as the filler amount increases, the mechanical properties decrease due to the poor compatibility between CaCO_3_ and PBAT. Furthermore, the high CaCO_3_ content will result in non-uniform dispersion and obvious agglomeration, resulting in decreased mechanical properties. The overall experimental result for the chosen CaCO_3_ filling amount is 30 wt.%, which not only maintains the performance of the composite material but also reduces its cost.

The tensile strength and elongation at break of PBAT composites filled with 30 wt.% modified CaCO_3_ were higher than those of unmodified CaCO_3_ due to the plasticizing effect of the coupling agent alkyl chains increasing the composite interfacial bonding energy; however, there is no interaction between the two phases of the matrix without the modifier, and it behaves as a brittle material. The results showed that adding the modifier significantly improved the mechanical properties of the composites. The tensile strengths of PBAT composites filled with modified CaCO_3_ (P7C3-d, P7C3-w, and P7C3-f) were increased by 3%, 14%, and 17%, respectively, compared with the unmodified CaCO_3_; and the corresponding elongation at break increased by 30%, 42%, and 44%, respectively. The relatively higher tensile strength and elongation at break of PBAT composites filled with gas–solid fluidization modified CaCO_3_ were attributed to the uniform distribution of the modifier molecules on the surface of CaCO_3_ during the gas—solid fluidized modification method, which resulted in better dispersion of the modified CaCO_3_ in the PBAT matrix and promoted the cross-linking of PBAT with the modified CaCO_3_, significantly improving the strength and toughness of the composite. As a result, the overall mechanical properties of the composites, such as strength and toughness, were significantly improved.

#### 3.2.4 Morphological analysis

**FIGURE 6:**
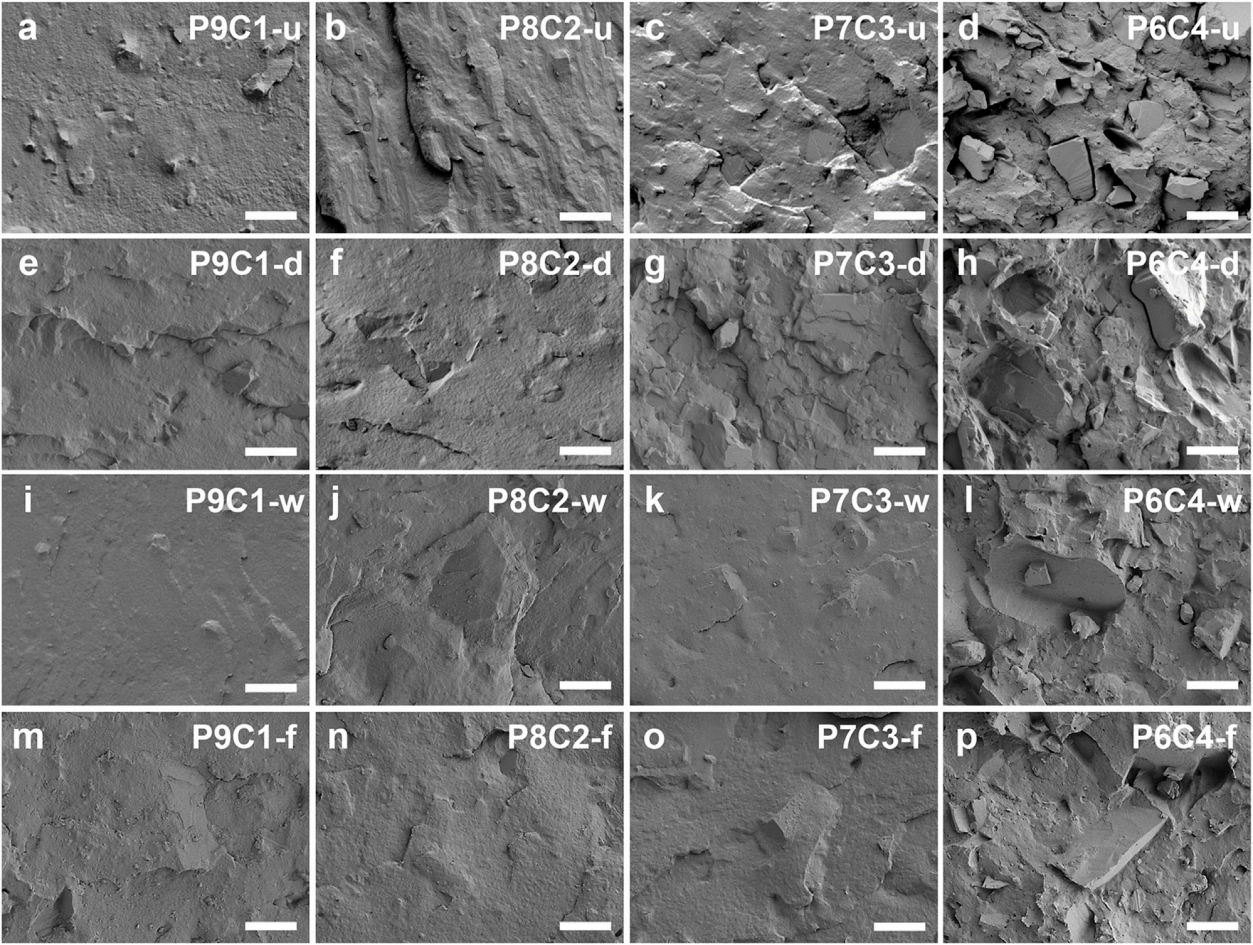
SEM images of PBAT composites filled with **(A)** 10, **(B)** 20, **(C)** 30, and **(D)** 40 wt.% of unmodified CaCO_3_; composites filled with **(E)** 10, **(F)** 20, **(G)** 30, and **(H)** 40 wt.% of dry-modified CaCO_3_; composites filled with **(I)** 10, **(J)** 20, **(K)** 30, and **(L)** 40 wt.% of wet-modified CaCO_3_; and composites filled with **(M)** 10, **(N)** 20, **(O)** 30, and **(P)** 40 wt.% of gas—solid fluidization modified CaCO_3_. The scale bar length is 20 µm.

Morphological analysis of the blends is frequently used to help us understand their compatibility and dispersion. Figures 6A–P depict scanning electron microscope (SEM) morphological images of PBAT composites filled with various CaCO_3_ contents and modification methods. According to the cross-sectional comparison in Figure 6, when the filler content was increased from 10 to 30 wt.%, CaCO_3_ was uniformly dispersed on the cross-sectional surface of the blends, and the surface was relatively flat, but when the filler content was increased to 40 wt.%, the tendency to form agglomerates was more obvious, and the interfacial compatibility was poor, resulting in poor performance of the composite. As a result, the optimal amount of CaCO_3_ filler preferred to be added is 30 wt.%. According to the longitudinal comparison in Figure 6, the general improvement in the apparent morphology of PBAT composites filled with dry-modified CaCO_3_ is due to poor dispersion uniformity and poor titanate modification effect by dry modification, which further leads to poor particle–matrix interfacial compatibility. In contrast, PBAT composites filled with gas—solid fluidization modified CaCO_3_ resulted in improved CaCO_3_ particle agglomeration, with large crystal size and low degree of agglomeration. Furthermore, the CaCO_3_ filler was well embedded in the PBAT matrix, and the two were more tightly bonded, resulting in improved composite performance.

## 4 Conclusion

A gas—solid fluidization method was proposed in this work to modify CaCO_3_. To generate biodegradable PBAT/CaCO_3_ composites, the modified CaCO_3_ filler was melt blended with PBAT and injection molded. The effects of various modification methods and filling amounts on the crystalline, mechanical, and rheological properties of PBAT/CaCO_3_ composites were investigated for comparison with dry and wet methods. The results showed that PBAT composites filled with gas—solid fluidization modified CaCO_3_ (at 30 wt.%) improved crystallinity (33.39%), and had excellent mechanical and rheological properties with uniform dispersion and interfacial compatibility. Therefore, the strategy proposed in this work provides an efficient modification method for preparing PBAT/CaCO_3_ composites that are low in cost and high in performance and can be applied more effectively in the field of ground film and other plastics.

## Data Availability

The original contributions presented in the study are included in the article/supplementary material, further inquiries can be directed to the corresponding author.
